# Profiling of circular RNAs in age-related cataract reveals circZNF292 as an antioxidant by sponging miR-23b-3p

**DOI:** 10.18632/aging.103683

**Published:** 2020-09-10

**Authors:** Shuqi Liang, Shengqian Dou, Wenfeng Li, Yusen Huang

**Affiliations:** 1 Medical College of Qingdao University, Qingdao, China; 2 State Key Laboratory Cultivation Base, Shandong Provincial Key Laboratory of Ophthalmology, Shandong Eye Institute, Shandong First Medical University and Shandong Academy of Medical Sciences, Qingdao, China; 3 Qingdao Eye Hospital of Shandong First Medical University, Qingdao, China; 4 Department of Medical Oncology, the Affiliated Hospital of Qingdao University, Qingdao, China

**Keywords:** age-related cataract, circRNAs, high-throughput sequencing, miRNAs, oxidative stress

## Abstract

Age-related cataract (ARC) is one of the major causes of visual impairment and reversible blindness worldwide. Accumulating evidence has revealed that circular RNAs (circRNAs) are involved in multiple regulatory processes in various ocular diseases. However, the expression profile, regulatory roles, and underlying mechanisms of circRNAs in ARC remain largely unknown. Herein we deep-sequenced circRNAs of anterior lens capsules from normal and ARC lenses, and detected 23,787 candidate circRNAs. Of these, 466 were significantly differentially expressed, and a higher correlation in down-regulated circRNAs between ARC and diabetic cataract was observed compared with up-regulated ones. Subsequent bioinformatics analysis disclosed that certain differentially expressed circRNAs participated in oxidative stress and apoptosis-related signaling pathways in ARC. Notably, the level of circZNF292 was significantly decreased, while miR-23b-3p was significantly increased in ARC. The target region prediction and dual-luciferase reporter assays proved that circZNF292 acted as a competitive endogenous RNA to regulate the expression of anti-oxidative genes through competing with miR-23b-3p. Our results indicate that circZNF292, a down-regulated circRNA in the anterior lens capsule of ARC patients, may be involved in resistance to oxidative damage and apoptosis of lens epithelial cells by sponging miR-23b-3p, providing a potential target for prevention and treatment of ARC.

## INTRODUCTION

Cataract is one of the most common eye diseases which result in visual impairment and reversible blindness [1, 2]. There are more than 20 million people with cataract-associated blindness worldwide [3]. Age-related cataract (ARC), a major type of cataracts, often starts at 45 to 50 years, and its incidence increases with age [4]. Currently, surgery intervention is the most effective therapy for cataracts [5], while a series of complications may affect vision quality [6]. Therefore, sustained efforts are needed to discover better solutions for prevention and treatment of cataracts.

In previous studies on the pathogenesis of ARC, oxidative stress was demonstrated to directly cause lens opacity and assumed to be a key factor during the development of cataracts [7, 8]. From the mechanism perspective, oxidative stress can induce the peroxidation of nucleic acids, lipids, crystalline proteins, and polysaccharides in cells, and activate signal transduction pathways and transcription factors, resulting in lens opacity of the eye [9–11]. Moreover, lens epithelial cells (LECs) are essential for maintaining metabolic stability and transparency of the whole lens [1, 12]. Apoptosis of LECs is a dominant cytological basis for the formation of cataracts except congenital cataract [13].

With the rapid advance of high-throughput sequencing technology, the role of circular RNAs (circRNAs), which emerge as vital regulators in various diseases, has drawn increasing attentions [14–17]. Unlike linear RNAs, circRNAs can form a covalently closed loop structure connecting the 3' and 5' ends, obtaining higher stability and more properties [18–21]. Conserved across species, they can express in a developmental stage- or tissue-specific manner, and be involved in multiple physiological processes and diseases [18, 20, 22–24]. CircRNAs are rich in miRNA response elements (MREs), which can communicate with target genes or co-regulate each other through competing to bind the shared miRNAs, thereby up-regulating the expression level of their target genes [25–27]. Therefore, as one of the non-coding RNAs with regulatory functions, circRNAs could antagonize microRNAs (miRNAs) through silencing target genes as miRNA sponges, and thus participate in the post-transcriptional regulation of host genes [25, 28, 29]. As reported in previous studies, gene regulatory networks such as circRNAs/miRNAs/mRNAs have provided us with a deeper understanding of the development of cataracts. For example, Liu et al. [30] proposed that the down-regulated expression of circHIPK3 could regulate the apoptosis of human LECs through the miR-193a/CRYAA axis in ARC. Our group disclosed that circKMT2E may play a role in the pathogenesis of diabetic cataract (DC) [31].

Moreover, numerous non-coding RNAs participate in oxidative damage during the cataract formation [32–34]. Cheng et al. [34] experimentally revealed that significantly up-regulated lncRNA H19 can repair oxidative damage to the lens of early ARC by regulating the miR-29a/TDG axis. However, it remains incompletely known whether circRNAs, members of the non-coding RNA family, have such a role in ARC.

In the current research, we explored the underlying mechanisms of circRNAs in the process of resisting oxidative damage by characterizing the interactions among circRNAs, miRNAs, and mRNAs in ARC for the first time. Among the differentially expressed circRNAs, circZNF292 was disclosed to act as a competitive endogenous RNA (ceRNA) of miR-23b-3p and be involved in reducing the oxidative damage to LECs by binding to anti-oxidative genes, thereby delaying the occurrence and development of ARC.

## RESULTS

### The landscape of circRNAs in ARC and normal tissues

**Figure 1:**
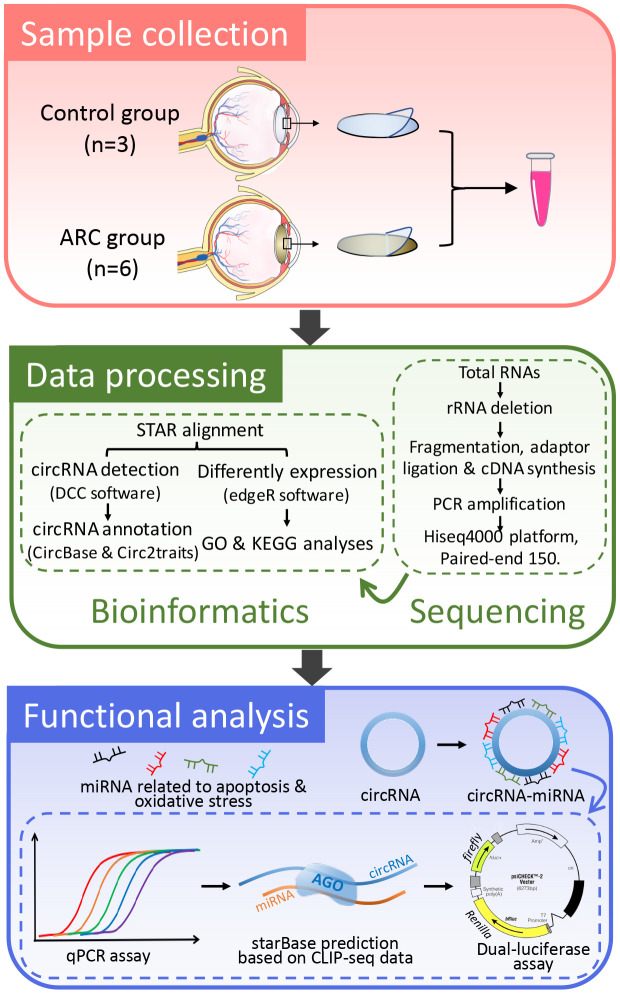
**A basic schematic diagram of our study.** This flowchart details the process of RNA-seq data acquisition and verification of the functional circRNAs in age-related cataract (ARC).

**Figure 2:**
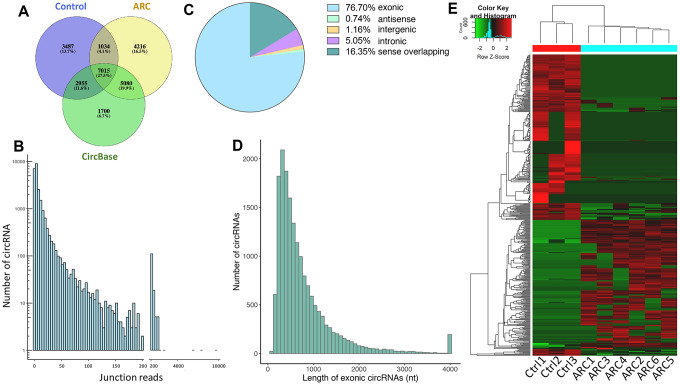
**Total circRNAs detected by RNA-seq in ARC and normal tissues.** (**A**) Overlaps of circRNAs identified in this study (ARC and the normal group) and CircBase. (**B**) The number of circRNAs and junction reads identified in ARC and normal tissues. (**C**) The genomic location of circRNAs. (**D**) The length distribution of exonic circRNAs. (**E**) The hierarchical clustering of circRNAs differentially expressed in ARC and normal tissues. Each row corresponds to a circRNA, and each column corresponds to a sample. The expression value is represented by a color scale. Intensity increases from green (relatively low expression) to red (relatively high expression).

To understand the regulatory roles of circRNAs in ARC, we first characterized the expression profile of circRNAs in anterior lens capsules. Briefly, we performed RNA sequencing (RNA-Seq) of ribosomal RNA (rRNA)-deleted total RNAs from normal (control group, 3 replicates) and ARC anterior lens capsules (ARC group, 6 replicates) (Figure 1A) on an Illumina HiSeq4000 platform, yielding about 100 million reads, which were mapped to the human reference genome (UCSC hg19) with STAR software [35]. A computational pipeline based on DCC software was used to identify circRNAs according to Ensembl transcriptome GTF file (Figure 1B). In total, 23,787 circRNA candidates were detected across all samples (unique junction reads ≥ 1) (Figure 2A, 2B), consisting of 17,345 circRNAs in the ARC group and 14,491 circRNAs in the control group (Table 1). Among them, 15,050 (63.3%) were overlapped with published ones obtained from circBase, while 8,737 (36.8%) circRNA candidates were newly identified (Figure 2A). For the functional annotation in the genome of circRNAs, 76.7% of them were located on protein-coding exons, but other circRNAs were aligned to introns, intergenic, antisense, and sense overlapping regions (Figure 2C). The length distribution of exonic circRNAs was shown with a median length of 560 nt (Figure 2D). Hierarchical clustering was then performed, demonstrating significant difference in the circRNA expression patterns between normal and ARC tissues (Figure 2E).

**Table 1:** Mapping statistics of total circRNAs.

**Sample**	**Raw reads**	**Q30**	**Clean reads**	**Clean ratio**	**Mapped reads**	**Mapped ratio**	**CircRNA number**
ARC1	100,528,824	92.99%	99,765,114	99.24%	91,788,422	92.00%	5,053
ARC 2	115,303,856	93.75%	114,395,206	99.21%	107,286,738	93.79%	6,029
ARC3	106,959,578	93.28%	106,220,576	99.31%	99,682,800	93.85%	6,124
ARC4	103,548,196	92.83%	102,057,780	98.56%	94,322,058	92.42%	5,489
ARC5	111,099,790	93.94%	110,795,950	99.73%	103,596,282	93.50%	7,381
ARC6	100,882,158	93.96%	99,826,302	98.95%	93,372,024	93.53%	6,708
Control1	80,127,844	93.74%	79,872,536	99.68%	73,434,472	91.94%	8,579
Control2	89,129,854	93.29%	88,683,664	99.50%	83,691,556	94.37%	4,934
Control3	111,189,984	92.20%	110,820,938	99.67%	100,434,050	90.63%	6,709

### Differentially expressed circRNAs in human ARC tissues

**Figure 3:**
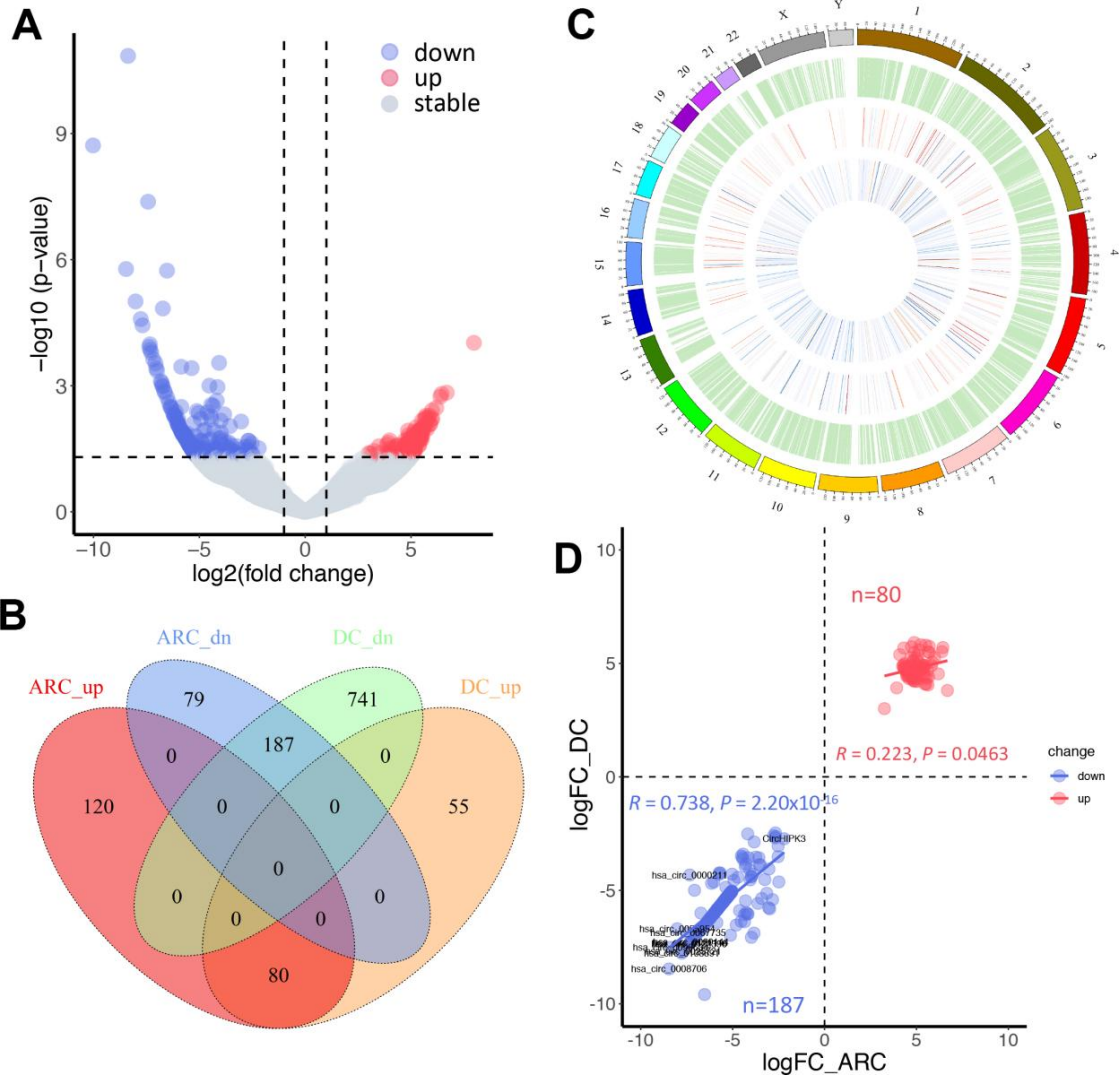
**Comparative analysis of differentially expressed circRNAs in ARC and diabetic cataract (DC) samples.** (**A**) A volcano plot of differential circRNAs in ARC. The vertical lines represent two-fold (log2 scaled) increased and decreased expression. The horizontal line shows P = 0.05 (-log10 scaled). Blue and red dots indicate circRNAs with statistically significant differential expression. (**B**) A Venn diagram shows differentially expressed circRNAs in ARC and DC samples. (**C**) Chromosomal distribution of expressed circRNAs. The outermost layer displays the location of the circRNAs in the human chromosomes. The inner circles from the outside to the inside show the expression distribution of all circRNAs in ARC and normal samples, differentially expressed circRNAs in ARC, and differentially expressed circRNAs in DC in turn. (**D**) Correlation of up- and down-regulated circRNAs in differential fold changes between ARC and DC samples (compared with normal tissues), and Pearson’s correlation coefficient R is presented.

The expression profiling of these circRNA transcripts revealed that numerous circRNAs were specifically expressed between ARC and normal tissues (Figure 2E). Therefore, we further analyzed the differentially expressed circRNAs in the samples. In this study, thresholds and criteria (fold-change ≥ 2, *P* < 0.05) were set to elect differentially expressed circRNAs (Figure 3A). Compared to the normal tissues, 466 significantly differentially expressed circRNAs were identified in the ARC tissues, among which 266 were down-regulated, and 200 were up-regulated. Since aging and hyperglycemia are key risk factors for cataract formation due to the deposition of reactive oxygen species (ROS) in LECs, causing cellular oxidative damage and even apoptosis [36–40], we carried out a further analysis of the significantly differential circRNAs in ARC and DC to narrow the scope for identifying circRNAs that play dominant roles in cataracts. As shown in Figure 3B, we surveyed the overlaps among up- and down-regulated circRNAs in ARC and DC, after which we characterized the chromosomal distributions of all circRNAs in ARC and normal samples, as well as differential circRNAs in ARC and DC, respectively. Furthermore, we calculated the correlation of differential fold changes between ARC and DC, finding a higher correlation in down-regulated circRNAs compared with up-regulated ones (Figure 3D). Hence we investigated the down-regulated circRNAs to hunt for circRNAs with regulatory roles in ARC formation.

### Functional analysis of the host genes of down-regulated circRNAs

**Figure 4:**
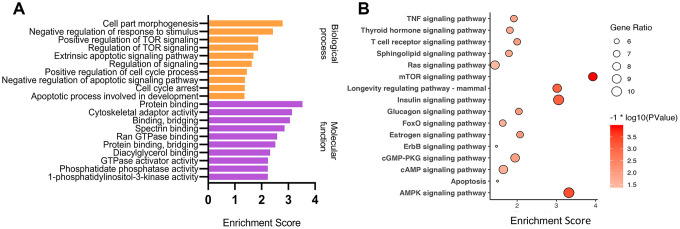
**GO and KEGG enrichment analysis of parental genes of differential circRNAs.** GO and KEGG enriched terms related to the ARC pathological process in an order based on the enrichment score [-log10 (P-value)] (**A**), and KEGG pathway analysis (**B**) of parental genes of differential down-regulated circRNAs in ARC tissues.

CircRNAs can act as miRNA sponges to regulate the expression of their host genes [41–43]. To better speculate the potential functions of down-regulated circRNAs in ARC, Gene Ontology (GO) and Kyoto Encyclopedia of Genes and Genomes (KEGG) enrichment analyses of their host genes were performed (Figure 4). In general, we focused on the enrichment terms and special functions which were related to the pathogenesis of ARC. In the biological process, metabolism was one of the main functions identified. The host gene functions were involved in many aspects of cell physiological functions such as growth regulation, stress response, and apoptosis. “protein binding”, “binding, bridging”, and “GTPase activator activity” in molecular functions of the GO enrichment analysis suggested that differentially expressed circRNAs may regulate cell proliferation or apoptosis by participating in cell signaling pathways. The KEGG pathway analysis showed that these parental genes of differentially expressed circRNAs were associated with the mTOR signaling pathway, AMPK signaling pathway, and longevity regulating pathway, which played a part in the oxidative stress and apoptosis process of cells [44–46]. These results provided some clues that down-regulated circRNAs may participate in the formation and development of ARC through various channels.

### Interaction network of circRNAs involved in oxidation resistance

**Figure 5:**
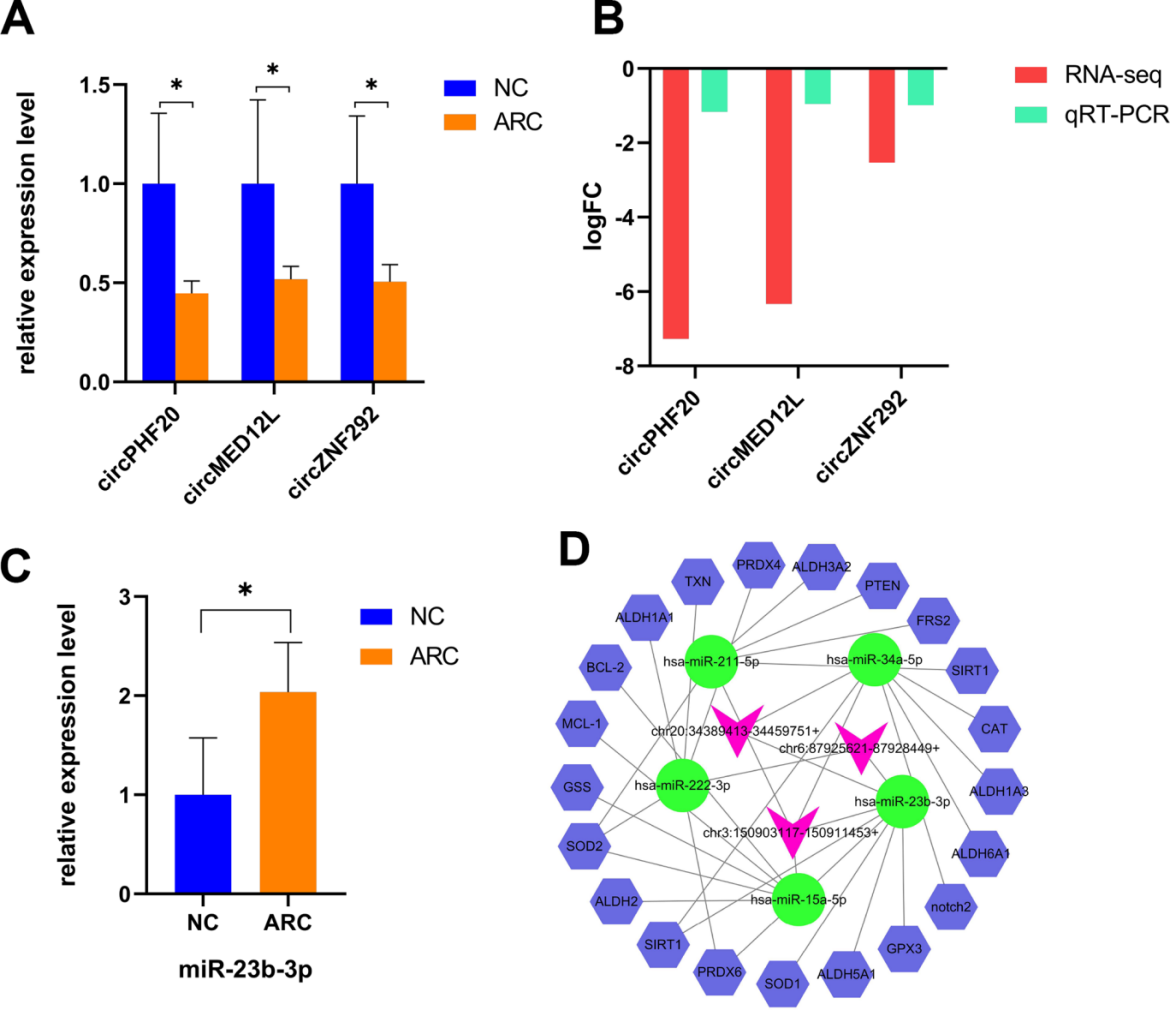
**Verification and the interaction network of selected circRNAs in ARC.** (**A**) qRT-PCR validation of candidate circRNAs in the control and ARC groups (*, P < 0.05). (**B**) The log2FC (fold change) of candidate circRNAs with significant difference by RNA-Seq and qRT-PCR. (**C**) Validation of miR-23b-3p in the normal and ARC groups. (**D**) A circRNA-miRNA-mRNA interaction map. The purplish red arrowheads represent circRNAs, the green ovals represent cataract-related miRNAs, and the blue hexagons represent the target genes related to oxidative stress. StarBase and TargetScan were used to predict the target mRNAs of these miRNAs, among which the oxidative stress related genes of miR-23b-3p are *GPX3*, *SIRT1*, *PRDX6*, *ALDH6A1*, and *SOD1*.

MiRNAs participate in the pathophysiological progression of various diseases, including cataracts [32]. However, the upstream regulations of miRNAs vary across tissues and diseases. CircRNAs can also serve as ceRNAs to prevent miRNAs from silencing their target genes [19, 25]. Many miRNAs, such as miR-34a-5p, miR-15a, and miR-23b-3p, have been reported to be significantly up-regulated in ARC lens tissues compared to normal tissues and exert important effects on cataract formation [47–51]. Based on the interaction between circRNAs and miRNAs, we identified candidate circRNAs which were down-regulated in ARC and interacted with miRNAs involved in oxidative stress and apoptosis for subsequent studies. General information of selected circRNAs is shown in Table 2. Then we used qRT-PCR to verify expression levels of the circRNAs, which showed a strong agreement to the patterns detected by RNA-Seq (Figure 5A, 5B). Coincidentally, we found that all candidate circRNAs were capable of interacting with miR-23b-3p, and thus we verified the expression of miR-23b-3p using qRT-PCR (Figure 5C). We also further predicted the target genes of miRNAs using TargetScan [52] and StarBase [53]. The antioxidative genes [54] were selected to construct a potential circRNA-miRNA-mRNA interaction network in the oxidative stress process of ARC (Figure 5D), representing the possible antioxidant effects of candidate circRNAs in detail.

**Table 2:** General characteristics of selected circRNAs.

**circBaseID**	**CircRNAID**	**logFC**	***P* Value**	**regulation**	**source**	**Best transcript**	**Gene Name**	**Catalog**	**predicted_sequence length**
hsa_circ_0060144	chr20:34389413-34459751+	-7.27035	0.000163	down	circBase	NM_016436	*PHF20*	exonic	1314 bp
hsa_circ_0122396	chr3:150903117-150911453+	-6.33003	0.003452	down	circBase	NM_053002	*MED12L*	exonic	651 bp
hsa_circ_0004058	chr6:87925621-87928449+	-2.52878	0.023552	down	circBase	NM_015021	*ZNF292*	exonic	370 bp

### Functional validation of circZNF292 via sponging miR-23b-3p in ARC

**Figure 6:**
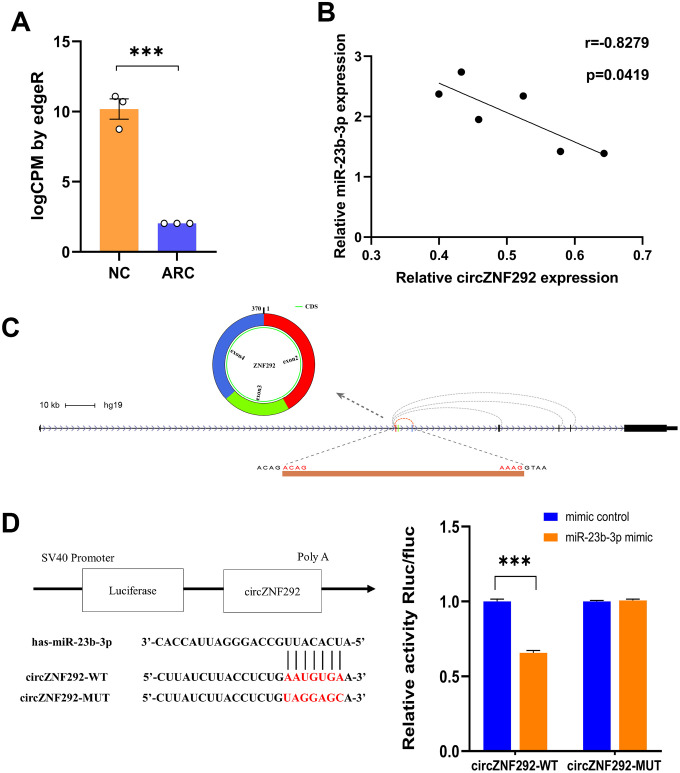
**General characteristics of circZNF292.** (**A**) RNA-Seq results of circZNF292 in the plasma from ARC patients. LogCPM by edgeR is the CPM value normalized by edgeR software and scaled by log2 (***, P<0.001). (**B**) The negative correlation of relative expression between circZNF292 and miR-23b-3p detected by qPCR (Pearson’s correlation coefficient R is presented and P < 0.05). (**C**) CircZNF292 was located between the second and fourth exons of its host gene. (**D**) Dual-luciferase reporter assay between circZNF292 and miR-23b-3p.

Circulating circRNAs are mostly fragments of apoptotic and necrotic cells released into the blood from different tissues. Among them, plasma circRNAs are considered as an ideal biomarker for disease detection due to their long-term stability even under extreme conditions [55]. Aqueous humor, transparent and colorless, is frequently used for fluid sampling in ocular examinations. It is derived from plasma, which can provide nutrients for the lens and clear metabolic wastes [56]. Our high-throughput sequencing results showed that circZNF292 was also dramatically down-regulated in the plasma of ARC patients (Figure 6A), so we selected circZNF292 as a functional biomarker for further research. The application of CLIP-Seq methods offers a reliable way to identify Argonaute (AGO) binding sites. From the database based on CLIP-Seq experimental techniques (StarBase), we found that circZNF292 can bind to miR-23b-3p via AGO proteins. CircZNF292 significantly decreased in patients with ARC compared with the controls, while hsa-miR-23b-3p significantly increased in patients with DC compared with the controls (Figure 5A, 5C). Accordingly, hsa-miR-23b-3p tended to negatively correlate with circZNF292 (Figure 6B). The interaction between circZNF292 and miR-23b-3p was then validated by dual-luciferase reporter assay, verifying the relationship between circRNAs and miRNAs. As shown in Figure 6D, the luciferase activity was significantly down-regulated by miR-23b-3p in the circZNF292-WT group compared with the control group, but was almost unchanged in the circZNF292-MUT group compared with its controls. Since circZNF292 proved to directly bind to miR-23b-3p, we hypothesized that circZNF292 can serve as a ceRNA by sponging miR-23b-3p and influence the progression of ARC.

## DISCUSSION

Multiple risk factors, like exposure to ultraviolet radiation, smoking, aging, metabolic disorders, and malnutrition, are linked to ARC creation [4]. Epidemiologic and experimental studies have indicated that the key contributors to ARC formation are exposure of the lens to oxidative stress [9–11] and LEC death [13]. Recent reports have shown that circRNAs are widely expressed in cataracts and play a part in the development of this eye disease [30, 31], but the role of circRNAs in oxidative stress remains unclear. The current study focused on the possible function of circRNAs in the repair mechanism of oxidative damage during cataract development.

In our study, we detected circRNA expression profiles in ARC in detail using second generation sequencing technology to explore their potential clinical value. A total of 466 significantly different circRNAs were detected, 92 of which were newly detected molecules. Similarly, in our sequencing data, circHIPK3 (chr11:33307959-33309057+, hsa_circ_0000284; logFC = -2.197, *P* < 0.05) was found to be significantly down-regulated in the ARC sample tissues [30]. Afterwards we analyzed significantly down-regulated circRNAs, as the correlation analysis results clearly indicated that they were more important for the study of cataracts.

Although the functions of most circRNAs in ARC are still not completely explored, it is possible to measure their molecular function and regulatory pathways by performing functional enrichment analysis of their parental genes. The expression changes in circRNAs and linear variants from the same genes have been reported to be largely correlated [18, 20, 29]. The possible functions of circRNAs are in line with their regulated and widespread expression. GO enrichment analysis indicated that different ARC circRNAs-derived genes could act by participating in signaling pathways. The KEGG pathway was enriched in ARC samples, after which several important pathways were identified, including AMPK and MTOR signaling pathways. AMP-activated protein kinase (AMPK), which is widely presented in eukaryotes, can be activated by lower intracellular ATP levels to regulate growth and metabolism, and has of late been disclosed to be associated with cellular processes [46]. By using melanoma mouse models, Kfoury et al. [45] demonstrated that the AMPK signaling pathway could suppress oxidative stress to promote survival of melanoma cells. Mammalian target of rapamycin (mTOR) is a serine/threonine protein kinase. Its signal pathway is closely related to cell proliferation and survival. P53 target genes (Sestrin1 and Sestrin2) have been reported to provide an important link among stress, p53, and the mTOR signaling pathway [44]. Therefore, we speculate that differential circRNAs may contribute to the formation of cataracts by participating in the apoptosis of LECs under oxidative stress.

CircRNAs, which have many binding sites with miRNAs, usually work as miRNA sponges to regulate the growth and development of organisms [18, 25, 29]. MiRNAs, a group of small non-coding regulatory RNAs of 21-23 nucleotides in length, can regulate gene expression through base-pairing with 3' untranslated regions (3’UTR) of their target mRNAs [57]. Involved in the intracellular processes of cataracts, including apoptosis, proliferation, activity, and oxidative damage [50], several miRNAs, like miR-34a-5p [47, 48], miR-15a [49], miR-23b-3p [51], and miR-211-5p [58, 59], could be specifically up-expressed in the lens and regulate the apoptosis or oxidative stress of LECs in the formation of ARC in experimental studies. Naturally, we selected circRNAs which were capable of binding these miRNAs based on databases (TargetScan, miRanda, and StarBase). Subsequently, qRT-PCR was conducted to verify expression of candidate circRNAs, presenting similar results with the verification.

**Figure 7:**
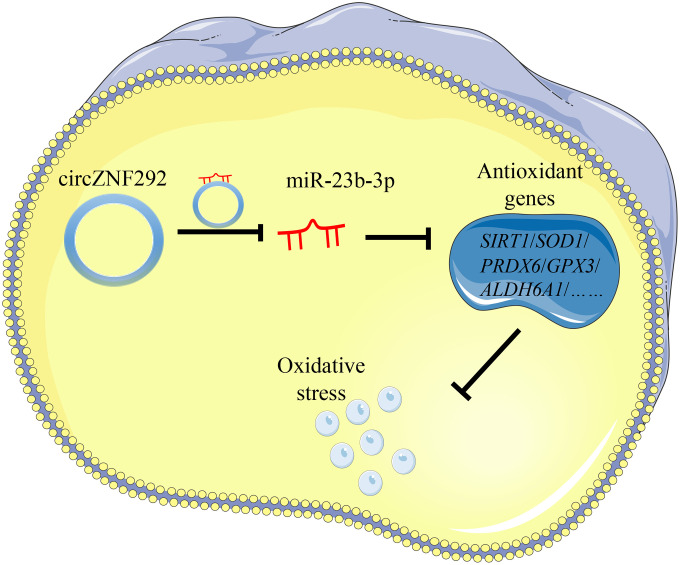
**General function pattern of circZNF292. This pattern outlines the process in which circZNF292 acts through the ceRNA network in ARC.**

To further explore the role of circRNAs in the pathogenesis of ARC, significantly down-regulated circZNF292 in the plasma and lens tissues of ARC was detected. CircZNF292 silencing has been reported to significantly decrease cell viability and increase apoptosis in Eca-109 cells [60]. Analogously, we hypothesize that circZNF292 may also fulfill such functions in LECs. Figure 7 briefly displays the process of exploring the function of circZNF292.

In this study, we observed that all the candidate circRNAs bound to miR-23b-3p, which seems to be an abnormal expression in cataractous human lenses and confirms the involvement of miR-23b-3p in the development of ARC. Zhou et al. [51] verified that miR-23b-3p can increase lens cell apoptosis by binding to Sirtuin 1 (SIRT1) under oxidative stress. Liu et al. [61] also indicated that miR-23b-3p can promote epithelial–mesenchymal transition of LECs by targeting sprouty2. We evaluated the significant up-expression of miR-23b-3p in the anterior lens capsules of ARC patients with its ceRNA network as the center, disclosing the expression levels of circZNF292 and miR-23b-3p were negatively correlated in human lens tissues. Our dual-luciferase reporter assay then supported that circZNF292 directly bound to miR-23b-3p and acted as a miR-23b-3p sponge to relieve the suppressive effect of miR-23b-3p target genes such as GPX3, SIRT1, PRDX6, ALDH6A1, and SOD1.

ROS-mediated oxidative stress is an important reason of LEC injury and apoptosis [11]. The physiological lens protective system decreases along with age. Long-term exposure of crystalline lens cells to oxidative stress can cause reduction in the lens repair mechanism and accumulation of oxidative damage, leading to cataract formation [7]. MiR-23b-3p plays a vital role in the occurrence of ARC. Among the predicted targets of miR-23b-3p, we found that many antioxidant genes interacted with miR-23b-3p. Subsequently, the interaction between miR-23b-3p and SIRT1 was verified in the SRA01/04(LEC) oxidative stress model, and miR-23b-3p was demonstrated to directly bind SIRT1 3'UTR to repress its expression [51].

SIRT1 is a special member of the sirtuin family and an important III NAD+-dependent protein deacetylase, which can regulate DNA stability and promote cell survival [62, 63]. Lin et al. [64] reported that the level of SIRT1 expression was negatively associated with patient age. Zheng et al. [63] also proved that SIRT1 can protect LECs from oxidative damage via the inhibition of the p53 pathway. SIRT1 helps maintain cellular homeostasis and has association with various ocular and other age-related diseases [63, 65]. To sum up, we propose that circZNF292 may resist oxidative damage and reduce apoptosis by sponging miR-23b-3p in human LECs. However, our study was only based on a comparison of transparent and clouded lenses. The specific role of circZNF292 in the pathogenesis of different types of cataracts remains unclear. Further studies on the relationship between circZNF292 changes and ARC formation are needed with age-matched human lenses in various cataracts and models in varying degrees of oxidative damage.

In conclusion, circRNAs had a certain correlation with the occurrence and development of ARC for their involvement in the oxidative stress and apoptosis of human LECs. After the differential expression analysis and function prediction of circRNAs in anterior lens capsules from ARC, down-regulated circRNAs associated with miR-23b-3p were selected for evaluation of the biological function and mechanism of circRNAs. CircZNF292, a significantly down-regulated circRNA involved in the oxidative stress-induced apoptosis process in LECs, was revealed as a potential antioxidant molecule in the pathogenesis of ARC.

## MATERIALS AND METHODS

### Sample collection

Anterior lens capsules were obtained from 18 patients (9 males and 9 females), aged 54 to 81 years (average, 67 years), who underwent ARC surgery at our institution. All these patients had no diabetes, hypertension or other ocular diseases. The capsules were removed by the same experienced surgeon (YH) during continuous curvilinear anterior capsulorhexis of cataract surgery. Moreover, nine transparent anterior lens capsules from healthy donor eyes were used as normal controls. All samples were immediately flash-frozen in liquid nitrogen before stored at -80 °C. Because the content of RNAs of a single lens anterior capsule was not adequate for RNA sequencing, three samples were combined for RNA extraction. Hence there were six replicates in the ARC group and three replicates in the control group (Figure 1A). For subsequent verification experiments, we collected the anterior capsule tissues from 15 patients with ARC and 6 normal donor eyes. The clinical characteristics of the study subjects are listed in Supplementary Table 1. The Lens Opacities Classification System III (LOCS III) was used for the assessment of lens opacity [66].

### RNA extraction, library preparation, and RNA sequencing

Total RNAs were extracted from frozen anterior lens capsule tissues using TRIzol reagent (Life Technologies, Carlsbad, CA, USA) in accordance with the instructions. The concentration of total RNAs was measured by a NanoDrop instrument (Thermo Fisher Scientific, Waltham, MA, USA), and the ratio of OD260/OD280 (between 1.8 and 2.1) was used as an indicator of RNA purity. The integrity of RNAs was also detected on the denaturing agarose gel electrophoresis according to the morphology of the 28S and 18S rRNA bands. Then library construction for sequencing was performed. Briefly, rRNAs in total RNAs were removed, and the purified RNAs were reverse-transcribed into complementary DNAs (cDNAs), adaptor ligated, and PCR amplified following the instructions of the TruSeq Stranded Total RNA Library Prep Kit (Illumina). RNA libraries were quality-controlled and quantified using the BioAnalyzer 2100 system (Agilent Technologies, Richardson, TX, USA) before sequenced on the Illumina HiSeq4000 Platform with a read length of paired-end 150 bp.

### NGS data processing and circRNA identification

The 3' adaptor sequences were trimmed with cutadapt software (v1.9.3) [67]. Clean reads were aligned to the reference genome of humans (UCSC hg19) with STAR (v2.5.1b) [35], after which circRNAs were detected and identified with DCC software (v0.4.4) according to Ensembl transcriptome (v75) GTF file. CircBase [68] (http://www.circbase.org) and circ2Trait [69] (http://gyanxet-beta.com/circdb/) were used to annotate the identified circRNAs.

### Differential gene expression analysis

The edgeR software (v3.16.5) [70] was used for RNA-seq data normalization and screening of differentially expressed circRNAs. Quasi-likelihood F test was performed to compare the difference in circRNA abundance between the ARC group and the control group. Significantly differentially expressed circRNAs were screened out at the cutoff of fold-change ≥ 2.0 with a *P* value < 0.05.

### Correlation analysis of differentially expressed circRNAs

Compared with the same control group, we performed RNA-seq on the human anterior capsule tissues of patients with ARC and DC [31]. R packets were used to analyze the correlation of significantly differentially expressed circRNAs between these two types of cataracts, with a correlation coefficient presented.

### GO and KEGG enrichment analyses

We performed GO (http://www.geneontology.org) and KEGG (http://www.genome.jp/kegg) enrichment analyses on the host genes of differentially expressed circRNAs. GO developed a structured, controlled vocabulary to describe genes and gene product attributes in organisms. The ontology covered molecular function, biological process, and cell component. Through the KEGG pathway analysis of host genes, we inferred the signaling pathways involved in circRNAs and their biological functions. The *P* value was obtained using fisher’s exact test, with a recommended cut-off at 0.05.

### Interaction network analysis of differentially expressed circRNAs

To clarify the role of differential circRNAs in cataracts, miRNA targets for circRNAs were predicted using target prediction software based on miRanda and TargetScan (Cloud-seq, Biotech, Shanghai, China). StarBase (https://web.archive.org/web/20110222111721/http://starbase.sysu.edu.cn/), which provides miRNA-target interaction prediction based on high-throughput CLIP-Seq data [53], was used to further screen circRNAs-miRNA pairs. Subsequently, we obtained circRNAs that interacted with ARC-related miRNAs. Then three significantly differential circRNAs with miR-23b-3p response elements were selected for further analysis.

### qRT-PCR verification of differential circRNAs and miRNAs

We used qRT-PCR to verify expression levels of differentially expressed circRNAs containing miR-23b-3p response elements. Total RNAs were extracted from lens tissues as described above, before cDNAs were synthesized using the SuperScriptIII Reverse Transcriptase Kit (Invitrogen). qRT-PCR was performed on the ViiA 7 Real-time PCR System (Applied Biosystems) using the qPCR SYBR Green Master Mix (Applied Biosystems). Specific primers for circRNAs were designed by primer 5.0 according to the sequence of the linear transcripts. Primer sequences of selected circRNAs and miRNA are shown in Table 3. Glyceraldehyde-3-phosphate dehydrogenase (GAPDH) and U6 were used as internal references, and the relative expression of circRNAs and miRNAs was calculated using the 2^-ΔΔCT^ method. Student’s t-test was used to determine significance for differences in circRNA expression with GraphPad Prism 8.0.

**Table 3:** Primer sequences of selected circRNAs and miRNAs.

**circRNA ID**	**primer type**	**gene sequence**
chr20:34389413-34459751+	Forward	GAACCGACTTCTCCCCTTGT
	Reverse	TCGTCTGTTAGGTGGATGCTT
chr3:150903117-150911453+	Forward	TGACCTCCTTCGCCACTTAC
	Reverse	CATAACAGCGTCACCACAGC
chr6:87925621-87928449+	Forward	AAGAGACTGGGGTGTGGAAA
	Reverse	TCTGAAGTTTTCCATTTCTCTGC
miR-23b-3p	Forward	GGGATCACATTGCCAGGGAT
	Reverse	CAGTGCGTGTCGTGGAGT
*GAPDH*	Forward	GGCCTCCAAGGAGTAAGACC
	Reverse	AGGGGAGATTCAGTGTGGTG
U6	Forward	CTCGCTTCGGCAGCACA
	Reverse	AACGCTTCACGAATTTGCGT

### ARC-related candidate circRNA analysis

To further elucidate the functional role of circRNAs, we selected circZNF292, which was markedly down-regulated in ARC plasma tissues, for further analysis. TargetScan and StarBase were applied to predict the mRNA targets of miRNAs. Based on a previous report [54], oxidative stress/apoptosis-related genes were selected. Then a map of the circRNA-miRNA-mRNA network was constructed using Cytoscape (3.7.1) software to demonstrate their interactions in ARC. UCSC Genome Browser (http://genome.ucsc.edu/cgi-bin/hgGateway) and circPrimer1.2 (http://www.bioinf.com.cn/) provided a visual representation of the circZNF292 composition and its location on the parental genes.

### Dual-luciferase reporter assay

A dual-luciferase reporter assay was performed to confirm the direct binding of miR-23b-3p and circZNF292. The wild-type and mutant sequences of circZNF292 were subcloned into multiple cloning regions (1640–1674 bp) of psiCHECK-2 luciferase reporter vectors (Progema). Then target-site containing plasmids, together with miR-23b-3p mimics or miRNA negative controls, were co-transfected into cells with Lipofectamine 2000 transfection reagent (Thermo Fisher). The fluorescence activities of firefly and *Renilla* were detected 48 hours after transfection according to the manual of the Dual-Luciferase Reporter Assay System (Progema), and the relative fluorescence value was calculated.

### Ethics statement

Anterior lens capsules used in this study were collected from patients undergoing surgery for ARC and healthy donor eyes provided by the eye bank of our institution. The study was performed in accordance with the tenets of the Declaration of Helsinki. The protocol was reviewed and approved by the Ethics Committee of Shandong Eye Institute. We informed the patients of the use of their specimens and obtained their consent.

## Supplementary Material

Supplementary Table 1
