# Supplementary material for: Profiling of circular RNAs in age-related cataract reveals circZNF292 as an antioxidant by sponging miR-23b-3p
Source: Aging (Albany NY). 2020 Sep 10;12(17):17271–87. doi: 10.18632/aging.103683 (PMC7521481; doi:10.18632/aging.103683)
Supplement: Supplementary Table 1 [file aging-12-103683-s001..pdf]

SUPPLEMENTARY TABLE

Supplementary Table 1. The clinical characteristics of the age-related cataract patients involved in this study.

Patient NO.	Age (y)	Gender	LOCS III Grade	Sample use
1	54	male	NO4C4P1, NC4	RNA-Seq
2	54	male	NO3C5P2, NC3	RNA-Seq
3	54	male	NO3C4P1, NC3	RNA-Seq
4	56	female	NO3C5P1, NC3	RNA-Seq
5	60	female	NO4C3P1, NC4	RNA-Seq
6	63	female	NO3C3P1, NC3	RNA-Seq
7	64	female	NO4C4P1, NC4	RNA-Seq
8	64	male	NO3C3P1, NC3	RNA-Seq
9	65	male	NO3C3P1, NC3	RNA-Seq
10	66	female	NO3C3P1, NC3	RNA-Seq
11	71	female	NO5C4P2, NC5	RNA-Seq
12	71	female	NO5C4P1, NC5	RNA-Seq
13	72	male	NO3C5P1, NC3	RNA-Seq
14	74	female	NO5C5P2, NC5	RNA-Seq
15	79	male	NO4C5P1, NC4	RNA-Seq
16	79	female	NO5C5P2, NC5	RNA-Seq
17	81	male	NO6C5P1, NC6	RNA-Seq
18	81	male	NO4C4P1, NC4	RNA-Seq
19	79	female	NO4C3P1, NC4	qRT-PCR
20	77	male	NO4C3P1, NC4	qRT-PCR
21	74	female	NO4C4P1, NC4/ NO4C4P1, NC4	qRT-PCR
22	70	male	NO3C3P3, NC3	qRT-PCR
23	68	male	NO5C4P1, NC5	qRT-PCR
24	67	female	NO3C3P1, NC3	qRT-PCR
25	67	male	NO4C3P1, NC4	qRT-PCR
26	65	female	NO3C2P1, NC3	qRT-PCR
27	65	female	NO3C3P1, NC3/ NO3C3P1, NC3	qRT-PCR
28	64	female	NO3C3P1, NC3/ NO3C3P1, NC3	qRT-PCR
29	63	male	NO3C5P1, NC3	qRT-PCR
30	63	male	NO5C4P1, NC5	qRT-PCR
31	62	female	NO5C3P1, NC5	qRT-PCR
32	59	male	NO3C4P1, NC3	qRT-PCR
33	57	male	NO5C3P1, NC5	qRT-PCR

Note: A total of 33 age-related cataract patients (36 eyes) in this study.
NC: nuclear color; NO: nuclear opalescence; C: cortical cataract; P: posterior subcapsular cataract.
